# Magnetic Moments and Electron Transport through Chromium-Based Antiferromagnetic Nanojunctions

**DOI:** 10.3390/ma11102030

**Published:** 2018-10-18

**Authors:** Marco Bragato, Simona Achilli, Fausto Cargnoni, Davide Ceresoli, Rocco Martinazzo, Raffaella Soave, Mario Italo Trioni

**Affiliations:** 1 Department of Chemistry, University of Milan, 20133 Milan, Italy; marco.bragato@unibas.ch (M.B.); rocco.martinazzo@unimi.it (R.M.); 2 Department of Physics, University of Milan, 20133 Milan, Italy; simona.achilli@unimi.it; 3 Consiglio Nazionale delle Ricerche, Istituto di Scienze e Tecnologie Molecolari and INSTM UdR di Milano, via Golgi 19, 20133 Milan, Italy; fausto.cargnoni@cnr.it (F.C.); davide.ceresoli@cnr.it (D.C.); raffaella.soave@istm.cnr.it (R.S.)

**Keywords:** antiferromagnetism, spintronics, electronic transport, DFT, *ab initio* calculations

## Abstract

We report the electronic, magnetic and transport properties of a prototypical antiferromagnetic (AFM) spintronic device. We chose Cr as the active layer because it is the only room-temperature AFM elemental metal. We sandwiched Cr between two non-magnetic metals (Pt or Au) with large spin-orbit coupling. We also inserted a buffer layer of insulating MgO to mimic the structure and finite resistivity of a real device. We found that, while spin-orbit has a negligible effect on the current flowing through the device, the MgO layer plays a crucial role. Its effect is to decouple the Cr magnetic moment from Pt (or Au) and to develop an overall spin magnetization. We have also calculated the spin-polarized ballistic conductance of the device within the Büttiker–Landauer framework, and we have found that for small applied bias our Pt/Cr/MgO/Pt device presents a spin polarization of the current amounting to ≃25%.

## 1. Introduction

In 1857 William Thompson first observed the phenomenon of magnetoresistance, which allows to significantly change the electrical resistance of a conductor by applying an external magnetic field. Much later, in 1988, Fert and Grünberg independently discovered what was later called giant magnetoresistance (GMR) [1,2]. What they observed is that a multilayered structure made by Fe/Cr/Fe offered a different resistance depending whether the magnetizations of the two iron slabs are parallel or antiparallel via a spin dependent interface scattering. GMR devices are generally composed by two ferromagnetic (FM) leads separated by a conductive layer. The resistance of such a device is easily tunable by modifying the magnetic orientation of one of the two layers, for example by applying an external magnetic field.

This discovery opened the way to *spintronics*, which aims at controlling and manipulating the electron magnetic moments and the spin polarized current. The research in spintronics led also to the discovery of tunnel magnetoresistance (TMR) [3], where electrons propagate thank to the tunnel effect, making the phenomenon strictly quantum-mechanical. These type of devices, called Mott devices, can achieve magnetoresistance ratios (defined as the ratio between the spin-polarized current and the total current) up to 20 times higher than normal GMRs: for these reasons they are going to be further investigated as promising future memory components.

In contrast to ferromagnetic substances, antiferromagnetic (AFM) materials are usually assumed to be invisible to common magnetic probes and insensitive to disturbing fields, due to their null macroscopic magnetization and strong AFM exchange interaction. AFM materials, however, have also some unique advantages, such as the absence of stray fields, the relative insensitivity to external magnetic fields, and the possibility to operate at very high frequency (typically in the terahertz regime) with little power losses. Not many AFM spintronic devices have been realized so far, and research in this area is now attracting significant attention [4,5]. The challenge is to manipulate the direction of the spins (their *quantization axis*) and detect a change of resistivity. This mechanism is called anisotropic magnetoresistance (AMR). The necessary requirements to enhance the magnetic anisotropy energy are (i) AFM order at room-temperature and (ii) large spin-orbit coupling (SOC).

In this paper, we address AFM spintronics from first principles density functional theory (DFT) calculations on a model system. In particular we study the electronic and transport properties of a thin layer of chromium between two non-magnetic (NM) leads, made of gold or platinum. We selected chromium because it is the only room-temperature AFM elemental metal, with a Néel temperature of 393 K. Contrary to Cr, Au and Pt are non-magnetic metals with large SOC. The idea is that the proximity between a heavy NM metal (of the 6th period) and a light AFM metal (of the 4th or 5th period) could result in a transfer of spin-orbit interaction from the former to the latter [6] and in a noticeable AMR. In addition to that, we also include a layer of insulating MgO, to increase the overall resistivity of the device, that would otherwise conduct too much current and overheat because of the Joule effect.

We anticipate that in the present case the spin-orbit transfer does not produce a noticeable AMR effect. However, the presence of the insulating buffer layer (MgO) effectively decouples the magnetic moment of Cr from the neighboring electrode and reduces the transfer of exchange-coupling between Cr and Pt, and to a lesser extent to Au. As a result, the proposed device displays an unexpected imbalance between the two spin channels and, as a consequence, a spin-filtering effect [7] on the current passing through the device.

The paper is organized as follows: in Section 2 we describe the computational details; in Section 3 we present the layout of the device. We then report its electronic, magnetic and transport properties, as a function of the choice of the heavy metal (Au vs. Pt) and of the absence/presence of the insulating layer. We also discuss the spin-polarized current I(V) and the magnetoresistance ratios. The effect of spin-orbit coupling in determining the properties of the systems studied is presented in Section 4. Finally, we propose our conclusions in Section 5.

## 2. Computational Details

**Figure 1:**
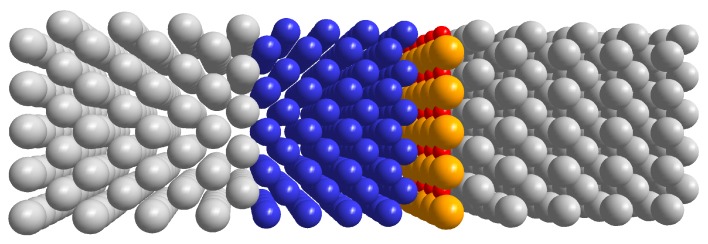
Schematic representation of the devices under investigation. Gold or platinum leads (grey) are intercalated with chromium (blue) and possibly an insulating layer of magnesium oxide (orange and red spheres, respectively).

In order to study the electronic and transport properties and their dependence on the spin orientation, we have considered the device reported in Figure 1. It is composed by three metal layers: two semi-infinite at the two ends (the leads, Pt or Au) and a finite one of Cr in the middle, with a varying number of mono-layers (ranging between 4 and 16). All the slabs are obtained by cutting the bulk along the (001) direction. Since Cr is *bcc*, while Pt and Au are *fcc*, and since the ratio between Pt/Au and Cr lattice spacings is close to $\sqrt{2}$, we rotated the Pt/Au lattices by 45${}^{\circ }$. In such a way we minimize the mismatch between the lattices. Besides metal/Cr/metal junctions, we have also studied systems with a MgO layer in order to simulate a more realistic device, where an insulating layer is inserted to increase its electrical resistivity. In these cases, the MgO layer has been inserted at just one of the Cr/Pt (or Cr/Au) contacts. MgO has a rocksalt lattice, with a spacing parameter comparable to the others, allowing for a good matching in the structure. The most stable arrangement is obtained when the oxygen atoms are directly above the metal ones, with the magnesium atoms occupying the hollow sites of the lattice. We considered electron transport along the direction perpendicular to the interfaces while the full periodicity is preserved in the transverse directions.

We performed ab initio calculations in the framework of the DFT [8,9] as implemented in SIESTA [10], a code that uses localized basis functions. We used the local density approximation (LDA) for the exchange-correlation energy functional described by Ceperley and Alder [11], and norm-conserving Troullier–Martin pseudopotentials [12]. We chose the LDA functional instead of the more popular GGA-family of functionals because, as already noticed in the literature [13,14], the magnetic moment of bulk Cr obtained with LDA calculations is much closer to the experimental value, as opposed to GGA values. Since in this work we are interested in modeling an AFM device, to rely on accurate atomic magnetic moments is mandatory.

We have used a standard double-ζ polarized basis set with an energy shift of 50 meV. The charge density was expanded with a cutoff of 350 Ry and the first Brillouin zone of the elemental metals was sampled with a 12 × 12 × 12 Monkhorst–Pack grid. To compute the transport properties we have used the Büttiker–Landauer formula [15,16,17], within the Green’s function formalism. This kind of approach considers only a ballistic regime of current flow and does not take into account inelastic scattering phenomena. However, since our nanojunctions are just a few nanometers wide, this is a reasonable approximation. We used the TranSIESTA code [18] for most calculations. Since the present version of TranSIESTA cannot deal with SOC, we have also used the GOLLUM code [19] to account for spin-orbit effects. We verified that in absence of spin-orbit, both codes yield transmission functions which are almost indistinguishable. If not otherwise stated, the results discussed in the following are obtained without inclusion of SOC.

## 3. Results and Discussion

### 3.1. Bulk Cr

Firstly we have investigated the electronic and magnetic properties of Cr, the only pure element that presents antiferromagnetism in its bulk phase at room temperature. Cr has an antiferromagnetic *bcc* structure with an experimental lattice constant $a_{\mathrm{lat}}=2.88$ Å [20] and a local magnetic moment of 0.5 Bohr magnetons (μB), as measured through an accurate neutron-diffraction study [21]. This value has to be compared with theoretical DFT estimates obtained within the LDA approximation, which range between 0.48 and 0.63 μB [13,14]. The magnetic moment strongly depends on the lattice constant: it increases with the size of the cell and vanishes for values below $a_{\mathrm{lat}}=2.85$ Å. Our theoretical and computational setup (see Section 2) yields a value of 0.48 μB for the local magnetic moment at the experimental lattice constant, in strong agreement with the experimental estimate.

### 3.2. Metal/Cr/Metal

We then considered a thin slab of chromium sandwiched between metal leads and cut through the (001) face. This plane exposes a layer with a ferromagnetic arrangement. After few tests in which we studied different thicknesses of Cr, we selected a slab with 8 layers of Cr as representative case. Obviously the Cr atoms are no more equivalent and the different distances from the interface generate a non trivial distribution of magnetic moments, approaching the bulk value in the middle of the slab.

We used two different 5*d* metals, namely Au and Pt, as semi-infinite leads. Our device is built in the following way: the (001) surface of the two metallic leads (both Pt or Au) are connected with the (001) surface of Cr, rotated by $45^{\circ }$ around the *z* axis. In such a way we obtain a match with the unit cell of Cr by expanding Pt of 4% with respect to its equilibrium lattice parameter, and compressing Au by less than 1%. Within this setup, we optimized the interlayer distance at the interfaces, obtaining values of 1.51 Å and 1.84 Å for Pt-Cr and Au-Cr, respectively.

The main difference between the two leads is represented by the electronic states at the Fermi level, which strongly influence the transport properties. In particular the Pt lead has got a significant density of *d* states at the Fermi level ($E_{F}$), while Au is a noble metal with a completely filled *d* shell. As a consequence, the degree of hybridization with the *d* states of the Cr slab will be different for the two metals, as will be discussed in the following.

**Figure 2:**
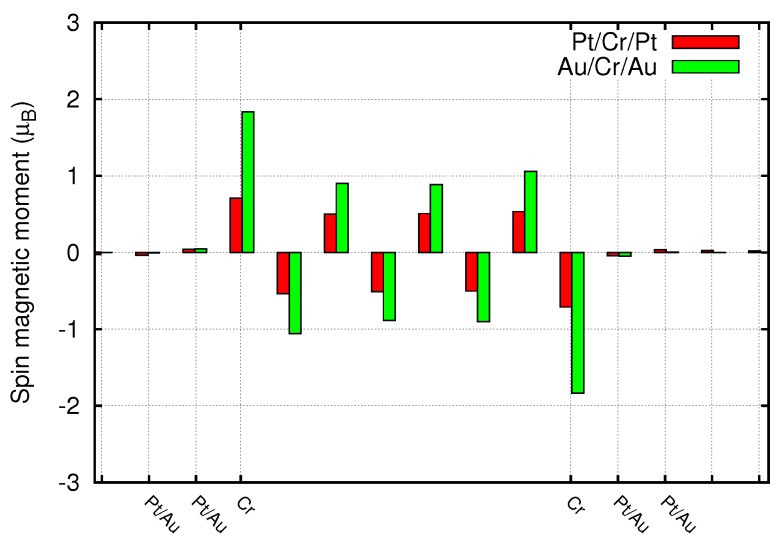
Spin magnetic moments along the junction constituted by Pt or Au leads and eight layers of Cr.

**Figure 3:**
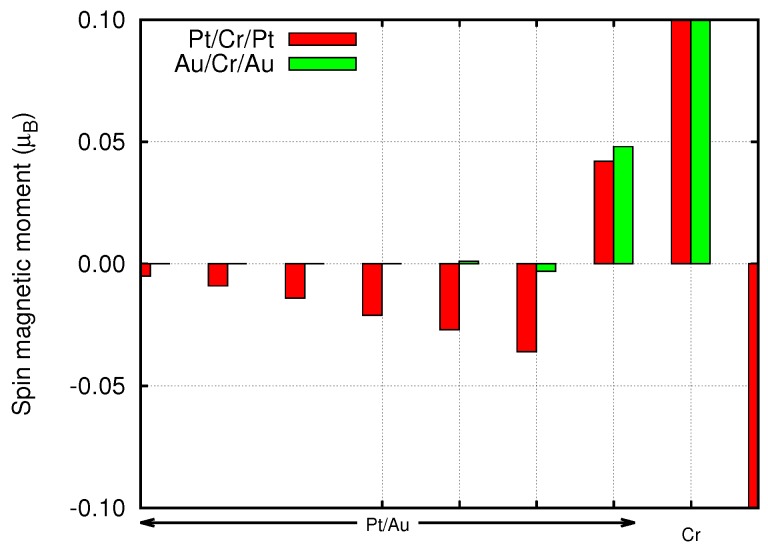
Spin magnetic moments at the first metal atoms at the Pt/Cr and Au/Cr interfaces.

The first result to be analyzed is the magnetic moment on any single Cr atom and, eventually, the moments induced in the first neighbors in the noble metals. Figure 2 reports the magnetic moments in μB. It can be immediately noted that the Au leads tend to enhance the magnetic moment in the Cr layers, differently from the Pt leads. This effect is explained considering the presence of the Pt *d* bands around the Fermi level, as those of Cr. The Cr hybridized states lose their magnetism taking some Pt character. Indeed in the case of thinner Cr slabs the magnetic moment on each atom is strongly quenched showing, for the four Cr layers case, values of 0.1–0.2 μB. This number raises increasing the number of layers up to eight, reaching the bulk value (≃0.5 μB) in the middle of the slab. For Au leads, which do not present *d* bands at the Fermi level, this hybridization is negligible and the magnetic moment of Cr atoms is enhanced due to the reduced dimensionality of the Cr sample. In both cases the Cr atoms in contact with the leads show the higher magnetization. The same arguments explain the presence of a tiny induced magnetization on the leads (see Figure 3), which is larger in Pt than in Au.

The electron transport properties in these nano-junctions can present a tiny spin polarization only due to the broken symmetry between the two spin components upon the application of a bias. To quantify this effect, we determined the electron transmission function, T(E) [15,16,17], which measures the probability that an electron of energy *E* coming from one of the electrodes is transmitted through the junction. The T(E) is strictly connected to the electronic current I(V) through the following equation:

(1) $$I\left(V\right)=\frac{2e}{h}\int_{-\infty }^{+\infty }T_{V}\left(E\right)\left[f\left(E-\mu_{L}\right)-f\left(E-\mu_{R}\right)\right]dE,$$

where f(E) is the Fermi-Dirac distribution, $\mu_{L}=E_{F}+V/2$ and $\mu_{R}=E_{F}-V/2$. Here we considered a null electronic temperature, and hence f(E) behaves like a step function. For small bias voltage (i.e., |V|<0.2 eV) it is possible to estimate the current without considering the non-equilibrium charge rearrangement, but simply applying a linear potential between the electrodes, while for larger bias voltages the transmission function should be computed self-consistently. However, devices working at low voltages are actively sought by experimentalists, to reduce their energy-consumption.

**Figure 4:**
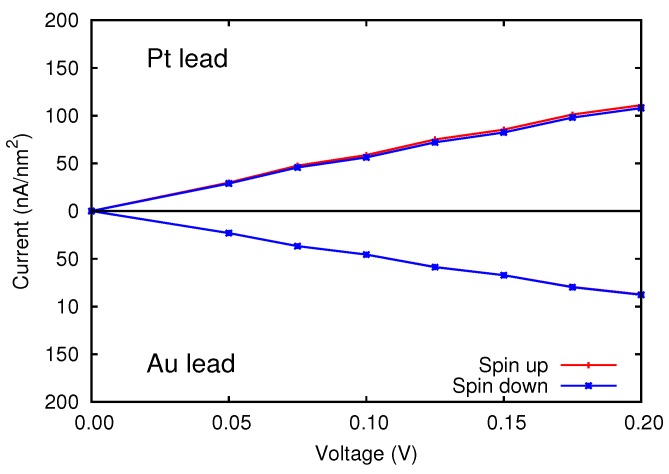
Spin resolved current of the symmetric devices, as a function of the applied bias voltage.

As a matter of fact, in our junctions the electronic current I(V) shows a very small spin polarization. This feature is negligible in Au/Cr/Au, and is very small also in Pt/Cr/Pt, never exceeding 1%, as shown in Figure 4. The inclusion of spin-orbit coupling does not alter appreciably our findings. We discuss its effects on the spin/orbital magnetic moments and on the transport properties in Section 4.

### 3.3. Metal/Cr/MgO/Metal

We then introduced a single spacing layer of MgO on one side of the junction. The purpose of MgO is two fold: the first is to effectively decouple Cr from Pt/Au reducing the hybridization; the second is to mimic the typical presence of a thin oxide layer in real junctions, which reduces the current density passing through the device.

**Figure 5:**
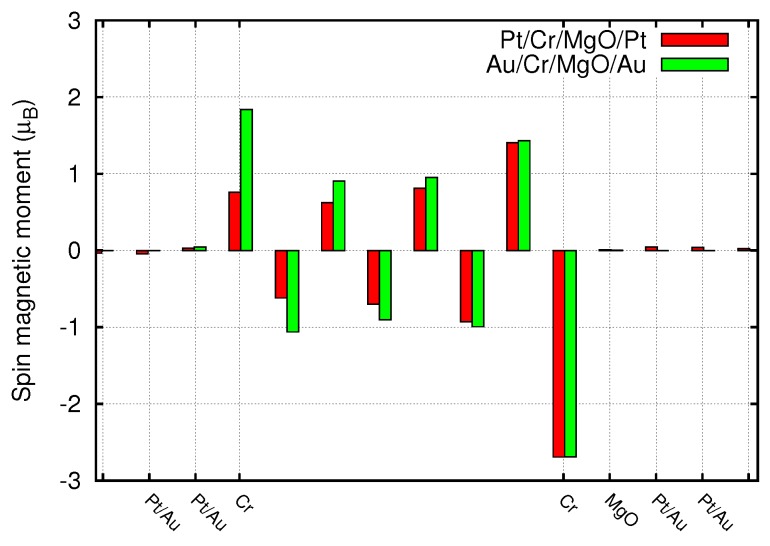
Spin magnetic moments along the junction constituted by Pt or Au leads plus 8 layers of Cr and a MgO monolayer.

Indeed, upon inserting one MgO layer in the nanojunction, the performance of our model device becomes much more intriguing. In Figure 5 we report the magnetic moment on each atom of the junction. Comparing these data with those of Figure 2, we note that the main consequence is a large enhancement of the magnetic moments of the contact Cr layer, which reach values close to 3 μB regardless of the chemical nature of the lead. The same effect is present also onto the neighboring Cr layer, though less relevant, while no significant variations can be envisaged in the inner ones. Therefore, these asymmetric devices develop a net spin polarization, due to the Cr/MgO interface. We verified that this spin polarization is basically independent on the Cr thickness, and is localized in a thin 2D region of space. This can be an advantage over the usage of 3D FM layers, whose magnetization generates stray fields that interact with neighboring junctions, thus preventing the down-scaling of the devices.

**Figure 6:**
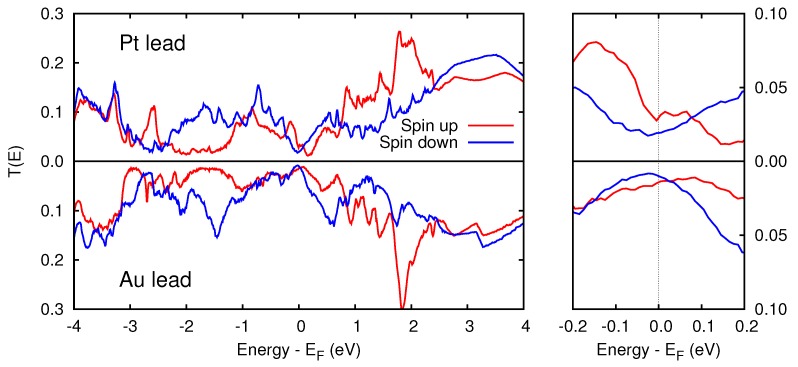
Left panel: spin resolved transmission function of the Pt/Cr/MgO/Pt and Au/Cr/MgO/Au devices, calculated with the Büttiker–Landauer formalism. Right panel: magnification of the region near $E_{F}$ is reported. The energy is referred to the Fermi level.

The spin polarization directly affects the transport properties of the device. In Figure 6 we report the transmission function T(E) for the two junctions, resolved in the two spin channels. It is likely that an asymmetric device will favor the electron transmission of one spin channel over the other, depending on energy. In the vicinity of the Fermi level, the Pt/Cr/MgO/Pt device shows a larger spin anisotropy with respect to the Au case. This effect is likely to be relevant when applying small external biases.

**Figure 7:**
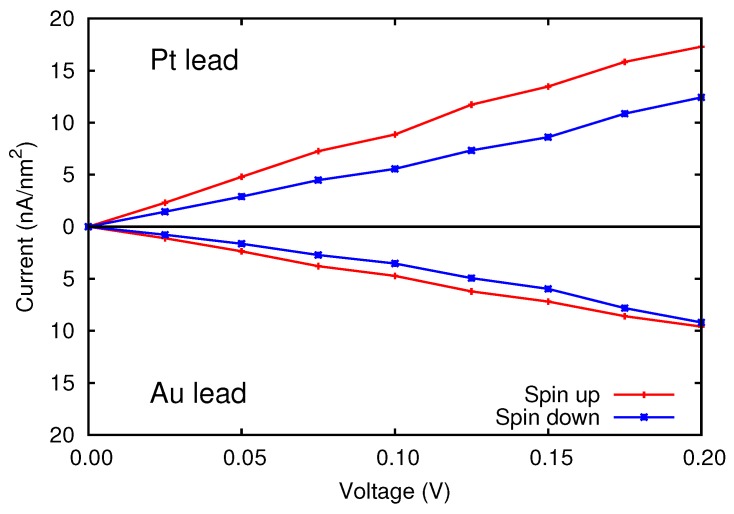
Spin resolved current of the asymmetric devices, as a function of the applied bias voltage.

As regards the I(V) curves, results reported in Figure 7 prove that for small voltages the Pt/Cr/MgO/Pt device is able to filter ≃25% of the spin down electrons, whereas the Au-containing junction is much less capable of filtering the spin. This can be explained again with the lack of hybridization between Cr 3d and Au 5d orbitals, at energies close to the Fermi level.

**Figure 8:**
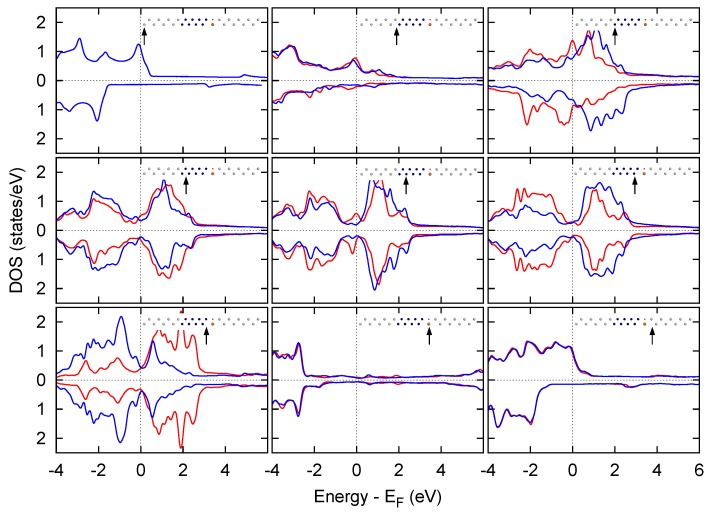
Spin resolved projected DOSs of asymmetric devices. The majority components are traced in red, and the minority components in blue. In each panel, the upper DOSs correspond to the Pt/Cr/MgO/Pt device, and the lower DOSs to the Au/Cr/MgO/Au one. Each panel reports the projection of the total DOS on a selected atomic layer, indicated by a black arrow.

In Figure 8 we reported the density of states (DOS) of the electrodes along with the spin-resolved projections of the total DOS of the device onto the most relevant atomic layers. It is easily recognizable that Au has no *d* states at the Fermi level, contrary to Pt. Furthermore, *d* states of metal atoms at the Cr/Pt interface are strongly hybridized, generating a peak in the majority component of the DOS of both atoms, centered at the Fermi level. No such effect is present in the inner Cr layers, whose projected density of states (PDOS) closely mimics a bulk-like behavior. Cr atoms at the Cr/MgO interface present the largest unbalance between up and down spin components. This issue resembles the behavior of the T(E) shown in Figure 6: it is likely that this layer is the main responsible for the spin filtering performance of the device. As expected, no relevant contribution to the DOS at the Fermi level can be ascribed to the insulating layer. The DOS of the metal atoms beyond the MgO is very similar to the bulk leads already from the first layer, notwithstanding the contact with the oxide.

More information on the nature of chemical bonding between the metal of the lead and chromium can be extracted from the analysis of crystal orbital Hamilton populations (COHP) [22]. This tool allows to decouple bonding between pairs of atoms in orbital contributions, and to establish the nature of bonding at selected energies. Since we are mostly interested in energy levels close to the Fermi level, responsible for electron transmission at low biases, we restricted our analysis to an energy range 0.4 eV wide and centered at $E_{F}$. In the case of Pt leads, the largely dominant contribution comes from interaction between *d* orbitals of Pt and of Cr, while *s*-*s* bonding is negligible. Conversely, in the case of Au leads the *d*-*d* bonding contribution between interface atoms is much smaller (about the half as compared to the case of Pt leads), and the *s*-*s* term plays a relevant role. This outcome is consistent with the results discussed above: electron transmission in the case of Pt leads depends mostly on hybridization between *d* orbitals of the lead and of chromium, while in the case of Au leads *d* orbitals are less important, and also *s* orbitals plays a relevant role. Moreover, this results are in agreement with the noticeable cohesion energy of the Pt/Cr interface, which amounts to 4.8 eV per surface unit cell, to be compared with the Au/Cr one which is instead only 2.6 eV.

## 4. Spin-Orbit Calculations

Upon switching on the spin-orbit interaction, no dramatic changes on the spin magnetic moments emerge. As spin-orbit coupling is included in the determination of the wavefunction, different solutions can be obtained depending on the orientation of magnetic moments with respect to the interfaces present in the device, i.e., parallel or perpendicular to them. Quite interestingly, in the case of Pt leads, when moments are parallel to the interfaces they reach absolute values about 25% larger than those obtained for the perpendicular orientation. Such effect is less relevant in the case of Au leads, a further indication that the bonding properties of Cr/Pt and Cr/Au contacts differ significantly from one another.

**Figure 9:**
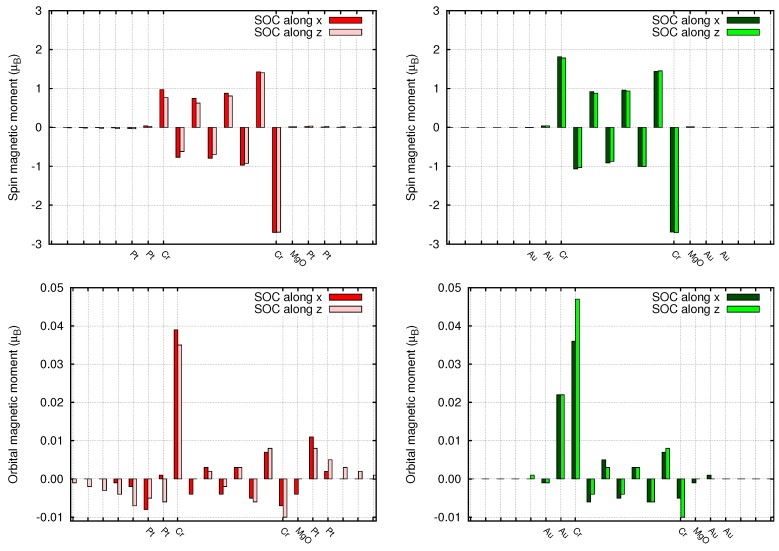
Spin (**top panels**) and Orbital (**bottom panels**) magnetic moments of the asymmetric devices, in presence of spin-orbit. Left panels: Pt/Cr/MgO/Pt. Right panels: Au/Cr/MgO/Au. The dark red and green bars denote the magnetic moments when the spin moments are oriented parallel to the interface (i.e., transverse to the transport direction). The light red and green bars are for the spins oriented perpendicular to the interface (i.e., parallel to the transport direction).

**Figure 10:**
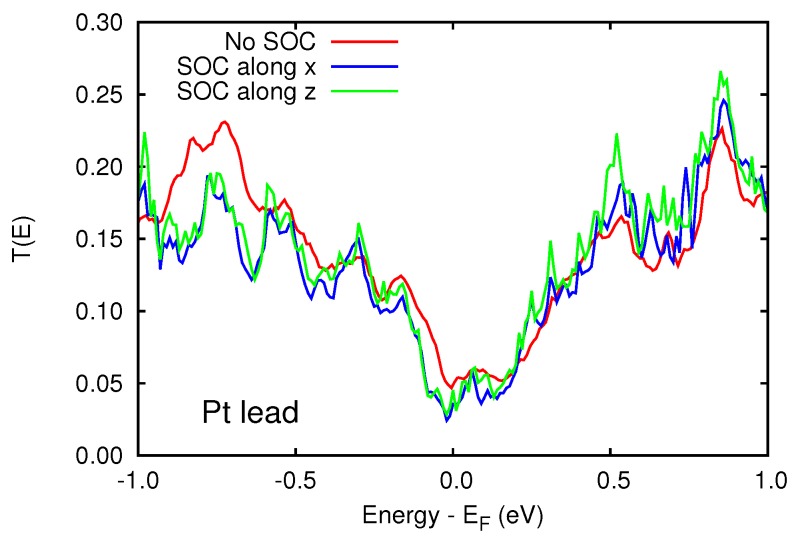
Transmission function of the asymmetric Pt lead device with and without spin-orbit interaction. In presence of SOC, we report T(E) as a function of the spin orientation with respect to the interfaces: *x* is parallel to the interface; *z* is perpendicular to the interface, i.e., parallel to the transport direction.

Since the spin-orbit coupling breaks time reversal symmetry, small orbital magnetic moments are generated by the local spin polarization. We calculated the orbital moments from the expectation value of the L operator on the atomic basis set. The orbital moments are in general two orders of magnitude smaller than the corresponding spin moments (see Figure 9) and are in general parallel to the spin moments. As a results of the reduced dimensionality, the orbital moments at the Au/Cr and Pt/Cr interface are less quenched and reach values of 0.4–0.5 μB. Similarly to the spin moments, the orbital moments decay quickly inside the left Au electrode, while they decay slowly inside the Pt electrode. Overall, these orbital moments, together with the spin moments, can be probed by x-ray magnetic circular dichroism (XMCD) experiments (see Ref. [23] for a recent review) and magneto-transport (Hall) measurements [24,25]. In order to have an anisotropy in the electronic current (AMR), it is necessary for the current to show a substantial spin polarization. In fact, due to the fine details of the change of the Fermi surfaces, as a function of global spin orientation, the current is expected to be different when the spins are aligned parallel or perpendicular to the interfaces. We calculated T(E) for the two orientations, with the GOLLUM code and we show them in Figure 10 in the case of Pt lead. Unfortunately, in the present case, the current anisotropy is basically vanishing. In the case of the Au/Cr/MgO/Au (not shown) this is expected, since also without SOC the current is not spin-polarized, and the absence of Au 5d states at $E_{F}$ further prevents any anisotropic effect. This is less expected upon replacing Au with Pt, and to fully explain this phenomenon, one should analyze the fine details of the spin-orbit split Fermi surface of Pt and Cr.

## 5. Conclusions

In conclusion, we have studied the electronic, magnetic and transport properties of a prototypical antiferromagnetic spintronic device, having Cr as active layer. Following the advice from literature, we sandwiched Cr between two non-magnetic metal leads (Pt or Au) with large spin-orbit coupling, to explore the possibility of a spin-orbit *transfer* from the electrodes to the AFM element. Contrary to expectations, we did not find any anisotropy in the magneto-transport properties (i.e., AMR effect) of such junctions.

Our results indicate that at the Au/Cr interface the magnetic moment of the latter metal is enhanced, while this does not occur at the Pt/Cr contact. This is due to the strong degree of hybridization between the electronic states of Cr and Pt, which is lacking in the case of Au leads. In both systems a residual spin polarization is found also on the lead atoms close to the interface with Cr.

To mimic the structure and the finite resistivity of a real device, we have inserted a buffer layer of insulating MgO between Cr and the heavy metal on just one side. Interestingly, we found that even a single MgO layer is sufficient to decouple the Cr magnetic moment either from Pt and Au, so that this asymmetric device develops a spontaneous spin polarization. Consistently, we demonstrated that the Pt/Cr/MgO/Pt device acts as a valuable spin filter, at least for small applied bias voltages.

Finally, the magnetic moment of the device is localized on a very thin region of space, close to the Cr/MgO interface, and the spin and orbital moments could be probed by x-ray magnetic circular dichroism experiments.

## Abbreviations

The following abbreviations are used in this manuscript:

AFM	antiferromagnetic
AHE	anomalous Hall effect
AMR	anisotropic magnetoresistance
COHP	crystal orbitals Hamilton populations
DFT	density functional theory
DOS	density of states
FM	ferromagnetic
GGA	generalized gradient approximation
GMR	giant magnetoresistance
LDA	local density approximation
NM	non-magnetic
PDOS	projected density of states
SOC	spin-orbit coupling
TMR	tunnel magnetoresistance
XMCD	x-ray magnetic circular dichroism
